# Effects of Soybean Meal Replacement on Growth Performance, Rumen Fermentation, Rumen Microorganisms, and Metabolites in Dumont Lambs

**DOI:** 10.3390/ani15213096

**Published:** 2025-10-24

**Authors:** Henan Lu, Hairong Wang, Boyang Li, Zenghao Lv, Shufang Li, Yuhao Xia, Lina Wang

**Affiliations:** 1 Animal Nutrition and Feed Science, Inner Mongolia Agricultural University, Hohhot 010018, China; lhena1117@163.com (H.L.);; 2 Key Laboratory of Animal Nutrition and Feed Science, Universities of Inner Mongolia Autonomous Region, Hohhot 010018, China

**Keywords:** rumen fermentation, microorganism, metabolomics, nitrogen, lambs

## Abstract

**Simple Summary:**

The price of soybean meal in China remains high and is mainly dependent on imports. Several factors have caused the price of soybean meal to fluctuate continuously, significantly impacting the sustainable development of China’s livestock industry. Therefore, this article utilises non-protein nitrogen (NPN), cottonseed meal and rapeseed meal as three relatively low-priced protein feed resources to replace part of the soybean meal for feeding Dumont lambs, exploring their effects on growth performance, rumen fermentation, and combined rumen microbial metagenomics and metabolomics to explain the reasons for the changes in phenotypic data. Compared with the soybean meal group, replacing 6.4% soybean meal with 1.5% urea significantly increased the rumen microbial protein (MCP) content, enhanced the degradation ability of fibres, facilitated energy metabolism, and provided a more efficient transportation capacity for nutrients. Replacing 4.3% soybean meal with 1% urea made the microbial composition and metabolic products of Dumont lambs closest to the soybean meal group and had higher MCP content and stronger linoleic acid metabolic ability. In conclusion, with 1% urea + 6.6% cottonseed meal + 5% rapeseed meal instead of all the soybean meal (19%), satisfactory results were obtained, enhancing the feed utilization efficiency and rumen fermentation, and optimizing the synergistic metabolic efficiency of carbon, nitrogen, and sulphur.

**Abstract:**

This study investigated the effects of replacing part of the soybean meal in the diet of Dumont lambs with urea, rapeseed meal, and cottonseed meal on their growth performance and rumen fermentation and combined rumen microbial metagenomics and metabolomics to explain the reasons for the changes in phenotypic data. Twenty-four healthy male Dumont lambs were divided into four groups: soybean meal group (T1, control group), group with 1.5% urea replacing 6.4% soybean meal (T2), group with 1% urea replacing 4.3% soybean meal (T3), and group with 1% urea + 6.6% cottonseed meal +5% rapeseed meal replacing all soybean meal (19%) (T4), following the principle of equal energy and nitrogen. Urea, rapeseed meal, and cottonseed meal have different degradation rates in the rumen, primarily stimulating arginine biosynthesis, sulphur metabolism, and carbon fixation in photosynthetic organisms through *Prevotella* genus mediation, thereby influencing the accumulation of metabolites such as 9,10-DiHOME, DG (PGJ2/a-15:0/0:0), isonicotinate and taxifolin, affecting rumen fermentation. Compared with the T1 group, the T2 group showed significantly increased ammonia nitrogen (NH_3_-N) and microbial protein (MCP) content (*p* < 0.01) and improved fructose and mannose metabolic capacity (*p* < 0.05). The T3 group showed a significant increase in total volatile fatty acids (TVFA) and MCP content (*p* < 0.01), which facilitated the absorption of subsequent nutrients. In the T4 group, different degradation rates of nitrogen resources and rapeseed meal + cottonseed meal contained abundant and complementary amino acids, which improved rumen fermentation, enhanced rumen microbial and metabolite diversity, and optimized the synergistic metabolic efficiency of carbon, nitrogen and sulphur. However, the specific mechanisms of post-rumen metabolism and absorption require further investigation.

## 1. Introduction

Mutton is of significant importance in consumption patterns due to its exceptional nutritional value and distinctive flavour, making the sheep industry a flourishing sector in China, resulting in increased demand for feed. The supply of soybean meal, one of the most important protein feed raw materials in the feeding industry, is especially insufficient due to resource competition and excessive demand, seriously restricting the healthy development of animal husbandry in China [1]. Therefore, the development and utilization of protein feed substitutes to reduce the utilization of soybean meal is one of the most important and difficult endeavours at present. As a new breed, the Dumont sheep is the hybrid offspring of the Dorper sheep and the Mongolian sheep. It was identified in 2022 as exhibiting a highly adaptable digestive system [2]. To fully exploit its production performance, the research team conducted some studies on the dietary composition in the early stage and found that this breed requires more concentrated feed to meet its energy and nitrogen demands compared to Mongolian sheep. Therefore, it has a higher demand for protein feed. In view of this, exploring the substitution of soybean meal is of greater importance. As a new breed, Dumont sheep are the offspring of crossbreeding Dorper and Mongolian sheep, officially recognized in 2022. They inherit the robust meat production genes from their Dorper sire lineage [2]. To maximize their production potential, our research team conducted early-stage feed formulation studies. We found that when fed identical diets, Dumont sheep consume an additional 2.38 MJ of feed daily and achieve an average daily gain 1.29 times that of Mongolian sheep [3]. This indicates superior appetite and digestive capacity for nutrient uptake. Whether this elevated production performance is underpinned by unique rumen microbiome characteristics warrants further investigation.

From the mid-20th century to the present, different nitrogen sources have been used in ruminant production to replace some conventional protein raw materials to reduce cost and increase efficiency, with one of the oldest being urea. Zheng et al. [4] conducted research showing that adding 1% urea to the feed can significantly increase the growth performance of 5-month-old fattening male Hu sheep. Slow-release urea can enhance rumen fermentation in heat-stressed dairy cows, alleviate heat stress in dairy cows, and reduce methane production [5]. However, previous research has primarily focused on the effects of urea alone on phenotypic indicators such as lamb growth performance and changes in rumen microbial communities. Few studies have attempted to combine urea with inexpensive protein feeds to comprehensively replace soybean meal, nor have they thoroughly investigated the systemic alterations in rumen microbial communities and their metabolites. For instance, Xu et al. demonstrated that 10 g of urea could substitute 130 g of soybean meal per kilogram of feed dry matter without adversely affecting growth performance or health in fattening lambs fed a high-concentrate diet but did not investigate alterations in rumen microbiota or metabolites [6]. Li et al. suggested that the rumen microbiome of sheep fed a low N diet with different urea supplementation are significantly different [7]. Research by Lu et al. suggests that urea supplementation affects microorganisms that carry nickel-dependent enzyme genes [8].

To account for the risk of ruminal acidosis, the use of urea in lambs is subject to restrictions. Moreover, there are alternative protein sources available here that can replace soybean meal. Cottonseed meal and rapeseed meal, as substitutes for soybean meal, can also alleviate the shortage of soybean meal supply due to their relatively low price and diverse sources while maintaining the same animal production performance [1,7,9]. Rapeseed protein exhibits a relatively balanced amino acid profile, with a high protein content, containing virtually no limiting amino acids. Compared to other meal products, rapeseed meal possesses the highest sulphur-containing amino acid content, followed by notably elevated levels of methionine and lysine [10]. Cottonseed meal is a byproduct of oil extraction from cottonseeds, serving as a valuable protein source for animal nutrition. Cottonseed meal contains 30–50% protein on a dry matter basis and is characterized by high amino acid concentrations, though it has relatively low lysine content [11]. In practical applications, cottonseed meal and rapeseed meal exhibit a complementary relationship. In actual production, when cottonseed meal serves as the primary protein source, supplementary additions of lysine and methionine are required. Combining cottonseed meal with rapeseed meal not only reduces the required methionine supplementation but also lowers the arginine-to-lysine ratio in cottonseed meal towards normal levels. This compensates for the insufficient arginine content in rapeseed meal, offering greater economic viability and safety in practical production. However, China’s abundant resources of cottonseed meal and rapeseed meal remain underutilised, with utilization rates of only 30–50%. The comprehensive utilisation rate of agricultural products stands at just 40%, significantly below the 90% achieved in developed nations. Furthermore, the development and utilisation of technologies for non-conventional protein resources (such as mixed meals) lag behind, resulting in severe resource wastage [12].

Compared to the isolated use of urea, the combined application of nitrogen sources such as urea, rapeseed meal, and cottonseed meal exhibits distinct degradation characteristics. When their ruminal degradation rates align with energy carriers (e.g., carbohydrates), they provide rumen microorganisms with synchronously available nitrogen and energy substrates, thereby promoting efficient MCP synthesis. The rational synchronised release of energy and nitrogen within the diet not only optimises the combination of various feed ingredients but also satisfies the carbon and nitrogen requirements of rumen microorganisms. This enables more complete utilisation of feed nutrients, thereby reducing waste and pollution while enhancing economic benefits [13].

Advancements in high-throughput sequencing technologies have provided new perspectives through omics approaches for analysing how different feeds influence rumen fermentation functions and growth performance indicators. Although previous studies have utilised omics approaches to investigate the role of urea in the rumen of ruminants, this study represents the first evaluation using three-month-old Dumont crossbred lambs as a model to assess the feasibility of reducing soybean meal and replacing it with urea, rapeseed meal, and cottonseed meal in high-performance breeds. This provides a theoretical basis for developing low-cost, highly efficient finishing diets for high-yielding crossbred lambs. In this manuscript, we replaced all soybean meal with urea, rapeseed meal, and cottonseed meal to investigate the feasibility of substituting low-cost nitrogen sources for soybean meal in Dumont lambs. Combining rumen microbial metagenomics and metabolomics, we analysed rumen microbial community composition and structural diversity, functional modules, rumen metabolites, and metabolic pathways. By applying rumen microbiome and metabolomics technologies, we elucidated the mechanisms underlying phenotypic data changes, providing theoretical support for reducing soybean meal dependency in practical production.

## 2. Materials and Methods

### 2.1. Animals and Diets

The experimental design and husbandry management adhered to the guidelines set by the ethical committee of the Institute of Animal Husbandry and Veterinary Medicine, Inner Mongolia Agricultural University, approved under (NND2022106). An animal experiment was conducted at the Hailiutu workstation of the Inner Mongolia Agricultural University. The experiment used a completely randomized experimental design. Twenty-four healthy 3-month-old male Dumont lambs with similar weights (23.29 ± 0.87) kg were selected for 15 d pre-feeding. Subsequently, 6 individuals per group were selected for 90 days of short-term fattening. All lambs were centrally allocated to group pens. The lamb house was cleaned thoroughly before the experiment, and the lambs were dewormed and immunized before entering the cage. Immunization protocols were implemented in compliance with university experimental policies. Diet was provided at 7:30 and 16:30. Lambs were allowed ad libitum access to both the mash feed and straw. Within each barn, lambs had free access to a water tank, which was cleaned once every two days, to ensure clean water quality. The experimental diet design followed the principle of isoenergy and isonitrogen in each group. The control group (T1) was fed a diet containing soybean meal at 19% as the main nitrogen source, and the other three experimental groups were fed a diet containing 1.5% urea instead 6.4% soybean meal (T2), 1% urea instead of 4.3% soybean meal (T3), 1% urea +6.6% cottonseed meal and 5% rapeseed meal instead of total soybean meal (19%) (T4). The ratio of cottonseed meal/rapeseed meal was 1.32:1, respectively (Table 1).

### 2.2. Sample Collection

#### 2.2.1. Sample Collection, Growth Performance, and Rumen Fermentation Parameter Measurement

All lambs were weighed before morning feeding at the beginning of the trial period at 0 d, 30 d, 60 d, and 90 d. The feed intake of test lambs was recorded daily. The recorded data were used to calculate growth performance indexes. The feed conversion ratio (FCR) was calculated with total feed consumption/total weight gain. On the 88th day of the trial period, six lambs from each group were selected for rumen fluid collection, three hours after the morning feeding. Rumen fluid was collected by a ruminal sampler (Zhejin Machinery and Equipment, Co., Ltd., Shijiazhuang, Hebei, China). The sampling catheter was inserted into the rumen through the mouth and oesophagus. The first 80 mL of ruminal fluid was collected and discarded. The remaining 200 mL of rumen fluid was immediately measured for pH with a pH meter (PHBJ-260F; INESA Scientific Instruments Co., Ltd., Shanghai, China). At the same time, the rumen fluid was dispensed into cryopreservation tubes, snap frozen in liquid nitrogen, and sent to the laboratory to be stored at −80 °C for the rumen fermentation experiment, subsequent rumen microbial metagenomics, and metabolomic sequencing. After returning to the laboratory, the rumen volatile fatty acid (VFA) was determined by a gas chromatograph (GC–7890B, Agilent Inc., Palo Alto, CA, USA). Capillary column: 30 m × 0.32 mm × 0.25 mm film thickness; column temperature = 110 °C; injector temperature = 180 °C; detector temperature = 180 °C). The specific method used was based on the study by Tao [14]. After that, the concentrations of NH_3_-N were determined as described by Broderick [15]. The content of MCP was determined by the Coomassie Brilliant Blue method, as described by Duo [16]; rumen microorganisms were obtained using differential centrifugation; and cell walls were broken using ultrasonic disruption. The proteins in the broken cell wall suspension were then stained using the Coomassie Brilliant Blue method and measured using an enzyme-labelled instrument (Multiskan FC, Thermo Fisher Scientific, Waltham, MA, USA).

#### 2.2.2. DNA Extraction, Metagenome Sequencing, and Metagenomics Data Processing

Total genomic DNA was extracted from rumen contents using FastPure Stool DNA Isolation Kit (MJYH, Shanghai, China). The concentration and purity of the extracted DNA were determined by SynergyHTX (BioTek Instruments, Winooski, VT, USA) and NanoDrop2000 (Thermo Fisher Scientific, Waltham, MA, USA), and the quality was verified by 1% agarose gel [17]. DNA extract was fragmented to an average size of about 350 bp using Covaris M220 (Gene Company Ltd., Tianjin, China) for paired-end library construction. Adapters containing the full complement of sequencing primer hybridization sites were ligated to the blunt end of fragments, then paired-end sequencing was performed. The data were analysed on the online platform Majorbio cloud platform (https://cloud.majorbio.com, accessed on 15 June 2023).

#### 2.2.3. Taxonomic and Functional Annotation from Rumen Metagenomes

Representative sequences of the non-redundant gene catalogue were aligned to the NR database with an e-value cutoff of 1 × 10^−5^ using Diamond [18] (https://github.com/bbuchfink/diamond, version 2.0.13, accessed on 8 August 2023) for taxonomic annotations. Cluster of ortologous groups of proteins (COGs) annotation for the representative sequences was performed using Diamond [18] (https://github.com/bbuchfink/diamond, version 2.0.13, accessed on 10 October 2023) against the eggNOG database, with an e-value cutoff of 1 × 10^−5^. The KEGG annotation was conducted using Diamond [18] (https://github.com/bbuchfink/diamond, version 2.0.13, accessed on 28 October 2023) against the Kyoto Encyclopedia of Genes and Genomes database (https://github.com/bbuchfink/diamond, version 2.0.13, accessed on 2 November 2023) with an e-value cutoff of 1 × 10^−5^. Carbohydrate-active enzyme annotation was conducted using *hmmscan* (http://hmmer.org/, version 3.1b2, accessed on 25 November 2023) against the *CAZy* database (http://www.cazy.org/, accessed on 30 November 2023) with an e-value cutoff of 1 × 10^−5^.

#### 2.2.4. Analysis of Rumen Metabolome

The rumen metabolome was analysed using a UHPLC-MS/MS method. The LC-MS/MS analysis of the sample was conducted on a Thermo UHPLC-Q Exactive HF-X system equipped with an ACQUITY HSS T3 column (100 mm × 2.1 mm i.d., 1.8 μm; Waters, Milford, MA, USA) at Majorbio Bio-Pharm Technology Co., Ltd. (Shanghai, China). The mobile phases consisted of 0.1% formic acid in water/acetonitrile (95:5, *v*/*v*) (solvent A) and 0.1% formic acid in acetonitrile/isopropanol/water (47.5:47.5, *v*/*v*) (solvent B). The flow rate was 0.40 mL/min, and the column temperature was 40 °C. The pretreatment of LC/MS raw data was performed by Progenesis QI (Waters Corporation, Milford, MA, USA) software, and a three-dimensional data matrix in CSV format was exported. The information in this three-dimensional matrix included sample information, metabolite name, and mass spectral response intensity. Internal standard peaks, as well as any known false positive peaks (including noise, column bleed, and derivatized reagent peaks), were removed from the data matrix, deredundant, and peak-pooled. The data matrix obtained by searching the database was uploaded to the Majorbio cloud platform (https://cloud.majorbio.com, accessed on 23 June 2023) for data analysis.

#### 2.2.5. Correlation Analysis

Correlation analysis between growth performance, rumen fermentation, rumen metagenome, and rumen metabolites was performed using Spearman’s rank correlation, with a *p*-value (*Spearman*’s rank correlation coefficient) <0.05 being considered as significant. All correlation analyses were performed using Spearman’s rank correlation, and a *p*-value <0.05 was considered significant. The correlation network was visualized using Cytoscape (Version 3.2.1, http://www.cytoscape.org, accessed on 4 May 2024). The correlation heat map was generated using the *R* program “pheatmap” package (https://www.r-project.org, version 4.4.8, accessed on 17 June 2024).

### 2.3. Data Statistics and Analysis

Analyses of the data on growth performance and rumen fermentation were conducted using a one-way ANOVA with SPSS 27 (IBM Corporation, Armonk, NY, USA). The *Tukey* method was used for multiple comparisons between groups. Significant differences were declared at *p* < 0.05. Highly significant differences were declared at *p* < 0.01. When *p* > 0.05, there were no significant differences. The microbial a-diversity of the experimental sample was assessed through QIIME2 (https://qiime2.org, Version 2024.5, accessed on 25 August 2024) software, which includes the Chao, Shannon, and Simpson indices. The degree of similarity of the sample microbial communities was evaluated based on the binary Jaccard distance using beta diversity in R (Version 1.6.2)., which primarily involved principal coordinate analysis (PCoA). Additionally, a Kruskal–Wallis test was conducted to determine the difference in rumen microbial phylum and genus with *p* < 0.05. Rumen microbial species were compared using linear discriminant analysis effect size (LEfSe), and significant differences were examined using an LDA score > 2 and *p* < 0.05 (LEfSe). The abundances of microbial metabolic pathways, modules, KEGG enzymes, and CAZymes were compared among the four groups using LEfSe, and significant differences were considered using an LDA score > 2 and *p* < 0.05.

## 3. Results

### 3.1. Effects of Urea, Cottonseed Meal, and Rapeseed Meal as Partial Replacements for Soybean Meal on Growth Performance and Rumen Fermentation of Dumont Lambs

The effects of urea, cottonseed meal, and rapeseed meal as partial replacements for soybean meal on growth performance are illustrated in Table 2. There was no significant difference in initial and final body weight between the four groups (*p* > 0.05). No significant difference was detected during the several daily feeding sessions (ADFI) of sheep and ADG (*p* > 0.05). The feed conversion ratio (FCR) of the T4 group was significantly lower than that of the T2 and T3 groups (*p* < 0.05).

As can be seen from Table 3, there was no significant difference in pH and A/P ratio among the four groups (*p* > 0.05). Compared with the T1 group, the T2 group exhibited significantly higher NH_3_-N and MCP contents (*p* < 0.01), as well as significantly lower isobutyric acid and valeric acid contents (*p* < 0.01). Compared with the T1 group, the T3 group exhibited significantly higher TVFA, butyric acid, valeric acid, and MCP contents (*p* < 0.01), as well as significantly lower acetic acid, isobutyric acid, and isovaleric acid contents (*p* < 0.01). Compared with the T1 group, the T4 group had significantly higher TVFA, MCP, and butyric acid contents (*p* < 0.01), as well as significantly lower isobutyric acid and isovaleric acid contents (*p* < 0.01).

### 3.2. Profiling of the Rumen Metagenome

Metagenome sequencing generated a total of 1,125,922,344 raw reads and 170,014,273,944 raw bases (bp), with 46,913,431 ± 713,377.55 reads (mean ± standard error of the mean [SEM]) per sample. After quality control, a total of 1,098,440,366 reads were retained, with 45,768,348.58 ± 699,610.12 reads per sample. After removing host genes, a total of 943,816,714 reads were retained, with 39,325,696.42 ± 663,763.70 reads per sample. After de novo assembly, a total of 6,366,976 contigs were generated (an N50 length of 1850.88 ± 145.98 bp), with 265,290.67 ± 21,912.20 reads per sample. The rumen metagenome consisted of 98.71% bacteria, 0.497% viruses, 0.26% archaea, 0.04% eukaryota, and 0.01% unclassified (Table A1).

### 3.3. Compositional Profiles of the Rumen Microbiome and Taxonomic Differences

**Figure 1:**
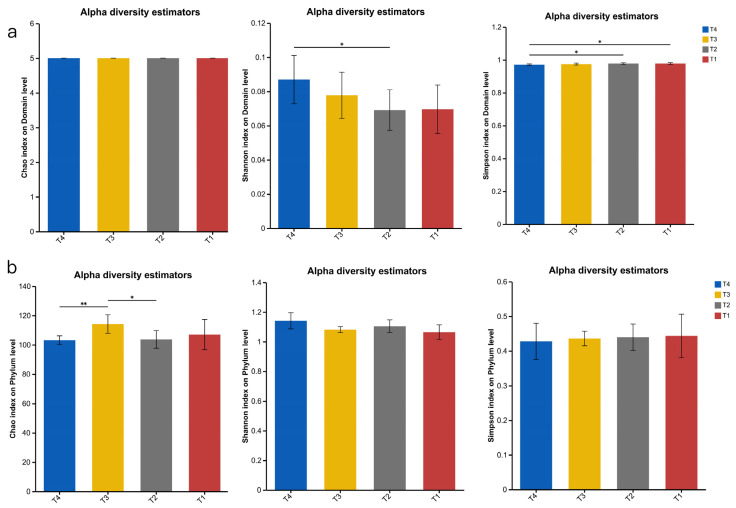

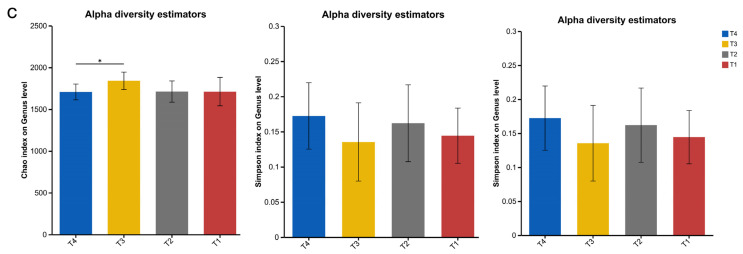
Microbial compositional a-diversity in the four groups. (**a**): Domain level. (**b**): Phylum level. (**c**): Genus level. T1: control group. T2: treatment with 1.5% urea as a replacement of 6.4% soybean meal in the diet of Dumont lambs. T3: treatment with 1% urea as a replacement of 4.3% soybean meal in the diet of Dumont lambs. T4: treatment with 1% urea + 6.6% cottonseed meal and 5% rapeseed meal as a replacement of total soybean meal (19%) in the diet of Dumont lambs. *: *p*-value < 0.05. **: 0.05 < *p*-value < 0.01.

The original gene set was analysed for a-diversity analysis, as shown in Figure 1. At domain level (Figure 1a), the Chao index was not significantly different among the four groups (*p* > 0.05), the Shannon index of the T4 group was significantly higher than that of the T2 group (*p* > 0.05), and the Simpson index of the T4 group was significantly higher than that of the T1 and T2 groups (*p* > 0.05). At the phylum level (Figure 1b), the Chao index of group T4 was significantly lower than that of group T3 (*p* < 0.01), and the Chao index of group T3 was significantly higher than that of group T2 (*p* < 0.05). At the genus level (Figure 1c), the Chao index of group T3 was significantly higher than that of group T4 (*p* > 0.05), with no other significant differences (*p* > 0.05).

**Figure 2:**
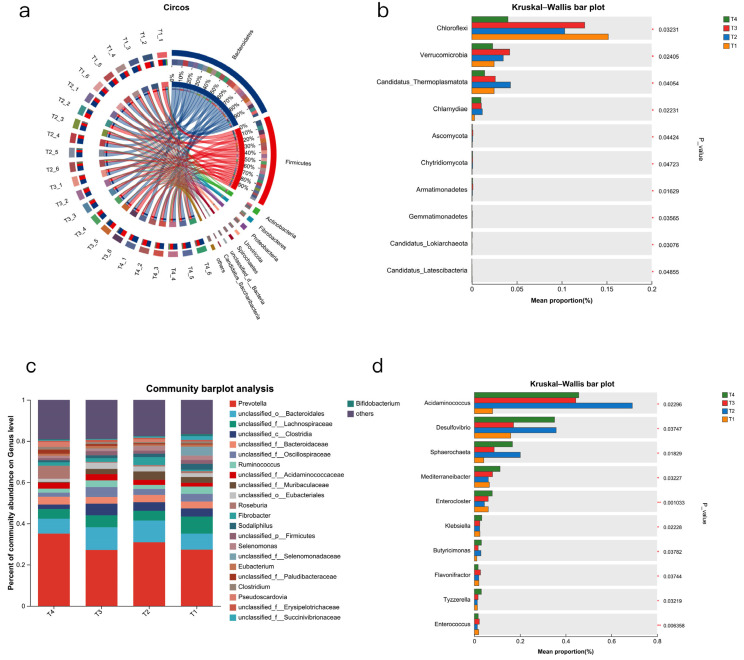
Microbial composition in four groups. A Circos diagram illustrating the relationship between sample and species at the phylum level, with (**a**) combined abundance = 0.01. (**b**) Community bar plot at the genus level, combined abundance = 0.01. (**c**) Comparison of microbial community phylum abundance with relative abundance ≥ 0.1% among four groups. (**d**) Comparison of microbial community genus abundance with relative abundance ≥ 0.1% among four groups. T1: control group. T2: treatment with 1.5% urea as a replacement for 6.4% soybean meal in the diet of Dumont lambs. T3: treatment with 1% urea as a replacement for 4.3% soybean meal in the diet of Dumont lambs. T4: treatment with 1% urea + 6.6% cottonseed meal and 5% rapeseed meal as a replacement for total soybean meal (19%) in the diet of Dumont lambs. *: *p*-value < 0.05. **: 0.05 < *p*-value < 0.01.

The microbial composition is shown in Figure 2. The dominant microbial phylum (Figure 2a) included *Bacteroidetes* (53.48 ± 1.25%) and *Firmicutes* (36.16 ± 0.002%). The dominant microbial genus (Figure 2c) included *Prevotella* (35.12 ± 0.19%) and *unclassified o Bacteroidales* (7.16 ± 0.44%).

For differential abundance comparison analysis at the phylum level (Figure 2b), the most abundant differentially represented microbes were *Chloroflexi* (*p* < 0.05), *Verrucomicrobia* (*p* < 0.05), and *Candiatus Themoplasmatota* (*p* < 0.05). The abundance of *Chloroflexi* in T4 was significantly lower than in T1 and T3 (*p* < 0.05). The T4 of *Verrucomicrobia* was significantly lower than T2 and T3 (*p* < 0.05), and T3 was significantly higher than T1 (*p* < 0.05).

At the genus level (Figure 2d), 100 genera exhibited significance, including 10 *Bacteroidetes*, 4 *Actinobacteria*, and so on. We listed the most abundant differentially represented microbes. The abundance of *Acidaminococcus* in T4 was significantly higher than in T1 (*p* < 0.05), the abundance of *Desulfovibrio* in T4 was significantly higher than in T1 and T3 (*p* < 0.05), and the abundance of *Sphaerochaeta* in T4 was highly significantly higher than in T1 and T3 (*p* < 0.05).

**Figure 3:**
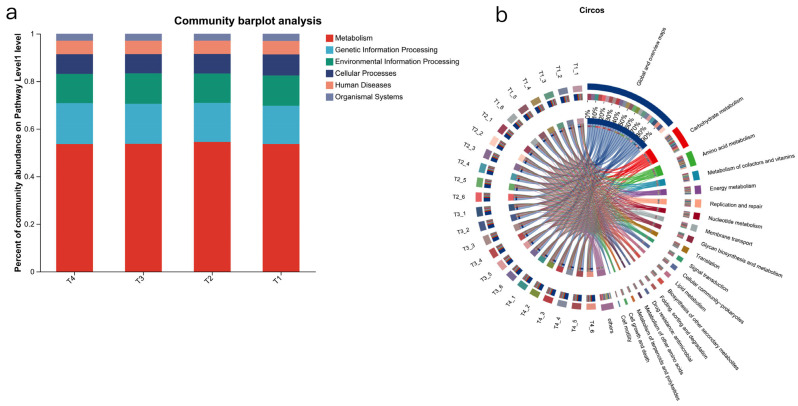

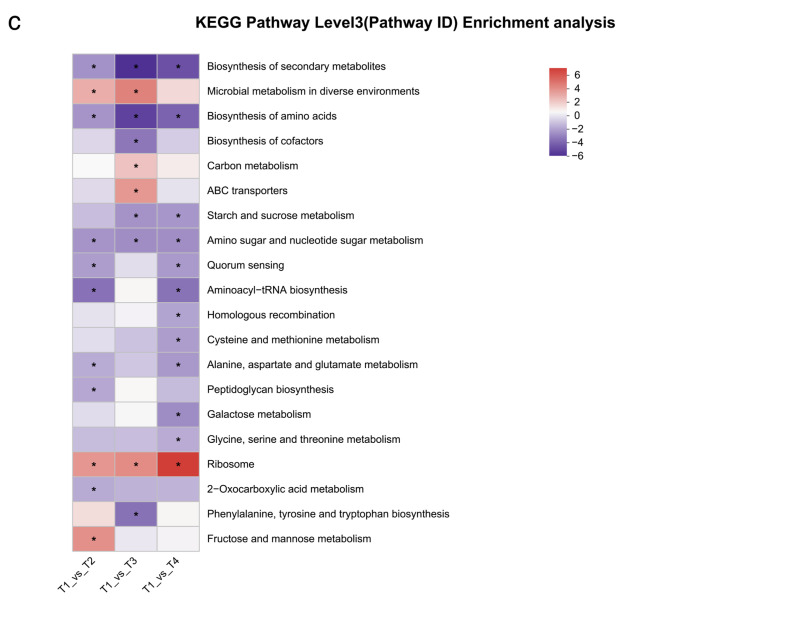
Function of rumen microorganisms. (**a**) Bar plot of rumen KEGG functions in level 1. (**b**) Diagram illustrating the relationships between samples (Circos plot) and KEGG level 2 functions; combined abundance = 0.01. (**c**) KEGG functional enrichment at level 3, which represents the most specific functional classification in KEGG, corresponding to specific biological metabolic pathways or molecular functions. T1: control group. T2: treatment with 1.5% urea as a replacement for 6.4% soybean meal in the diet of Dumont lambs. T3: treatment with 1% urea as a replacement for 4.3% soybean meal in the diet of Dumont lambs. T4: treatment with 1% urea + 6.6% cottonseed meal and 5% rapeseed meal as a replacement for total soybean meal (19%) in the diet of Dumont lambs. *: *p*-value < 0.05.

**Figure A2:**
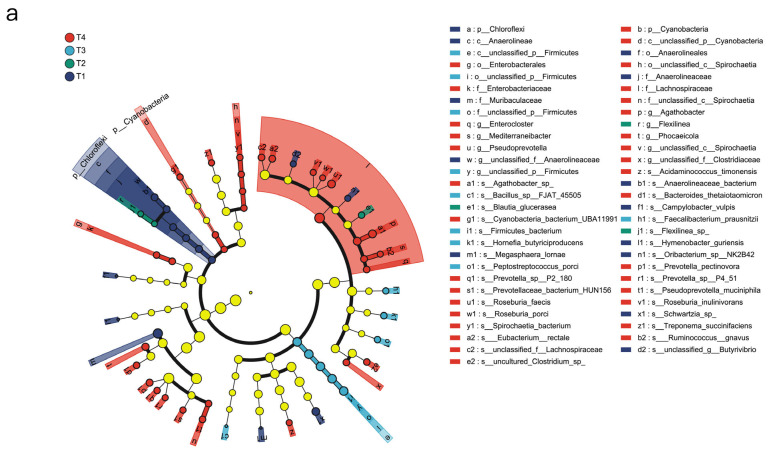

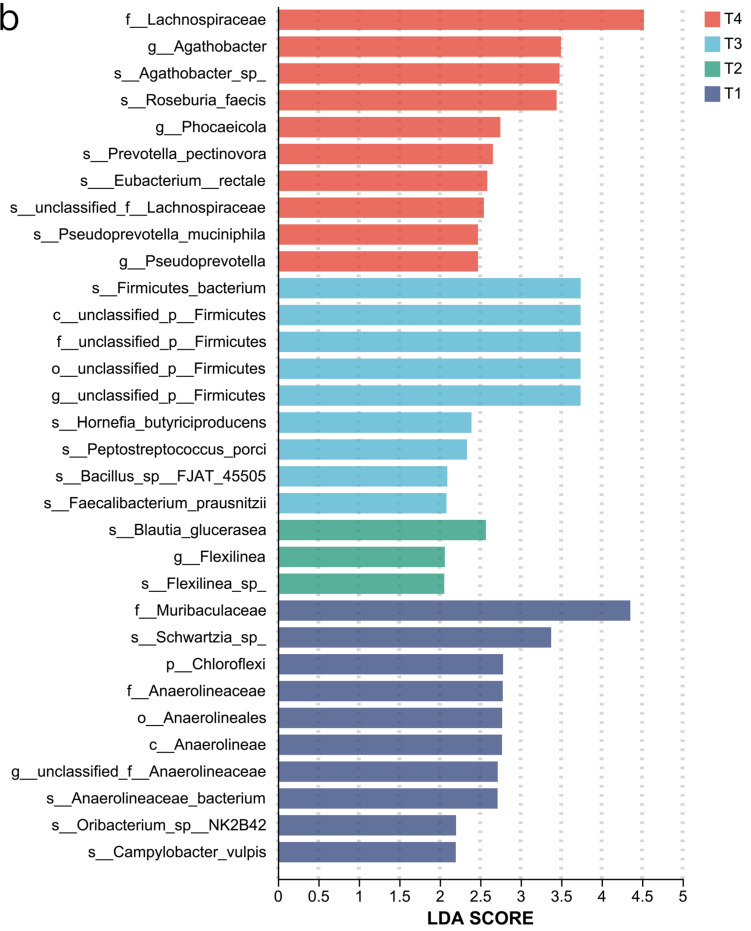
LEfSe analysis of bacteria of sheep in four groups. (**a**) Cladogram of LDA scores. (**b**) Bar graph of the LDA value distribution. T1: Control group. T2: Treatment with 1.5% urea instead 6.4% soybean meal on Dumont lambs. T3: Treatment with 1% urea instead 4.3% soybean meal on Dumont lambs. T4: Treatment with 1% urea + 6.6% cottonseed meal and 5% rapeseed meal instead total soybean meal (19%) on Dumont lambs.

Bacteria make up the largest portion of the rumen. LEfSe was used to analyse differential bacteria abundance, as shown in Figure 3a. The bar graph shows the LDA value of different bacteria (LDA > 2) (Figure A2a).

The greater the LDA score, the greater the impact of species abundance on the differences among groups. The bacterial strains in the T2 group that showed significant differences were *s__Blautia_glucerasea*, and *g__Flexilinea s__Flexilinea_sp_*. The bacterial strains in the T1 group that showed significant differences were *f__Muribaculaceae s__Schwartzia_sp_*, *p__Chloroflexi*, et al. The bacteria strains in the T3 group that showed significant differences were *s_Firmicutes_ bacterium*, *c__unclassified_p__Firmicutes f__unclassified_ p__Firmicutes*, and *o__unclassified_p__Firmicutes g__unclassified_p __Firmicutes*. The bacterial strains whose abundance was significantly affected in the T4 group were *f__Lachnospiraceae*, *g__Agathobacter*, *s__Agathobacter_sp_*, *s__Roseburia_faecis*, et al. (Figure A2b).

### 3.4. Functional Differences of Rumen Microorganisms in Dumont Sheep

The functions of the rumen microbiome were determined based on the Kyoto Encyclopaedia of Genes and Genomes (KEGG) database. For the KEGG profiles, 131 endogenous third-level pathways were considered as rumen microbial metabolic pathways. These pathways belonged to six first-level categories, including “*Metabolism*”, “*Genetic information processing*”, “*Environment information processing*”, “*Cellular processes*”, “*Human Diseases*”, and “*Organismal Systems*” (Figure 3a). At the second level, 35 categories were observed, with “*Global and overview maps*” “*Carbohydrate metabolism*”, “*Amino acid metabolism*”, “*Metabolism of cofactors and vitamins*”, and “*Energy metabolism*” being the most abundant (Figure 3b).

When the identified KEGG pathways were compared, seven third-level pathways were significantly enriched in T2, six third-level pathways were significantly enriched in T3 (*p* < 0.05), and three third-level pathways were significantly enriched in T4 (*p* < 0.05). Furthermore, KEGG enrichment analysis was conducted at the level 3, and it was found that the T2 group was mainly significantly enriched in “*Fructose and mannose metabolism*” (*p* < 0.05), the T3 group was mainly significantly enriched in “*ABC transporters*” (*p* < 0.05), and the T4 group was mainly significantly enriched in “*Ribosome*” (*p* < 0.05).

**Figure A3:**
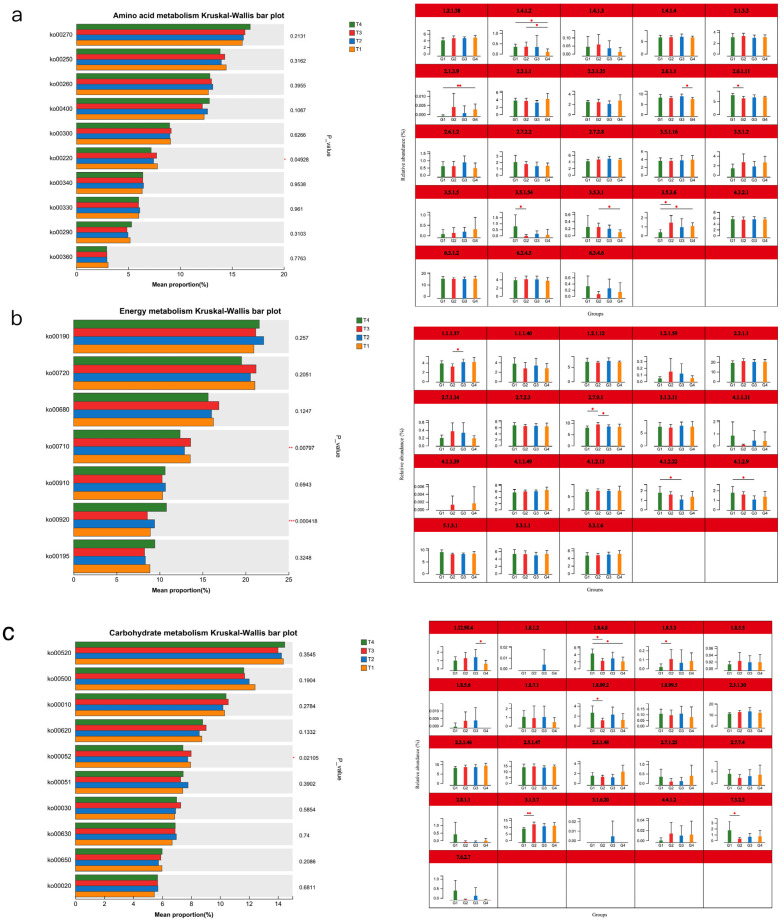
(**a**) Different ko in Carbohydrate metabolism and the expression of enzymes in the above three metabolic processes. (**b**) Different ko in Amino acid metabolism and the expression of enzymes in the above three metabolic processes. (**c**) Different ko in energy metabolism and the expression of enzymes in the above three metabolic processes. T1: Control group. T2: Treatment with 1.5% urea instead 6.4% soybean meal on Dumont lambs. T3: Treatment with 1% urea instead 4.3% soybean meal on Dumont lambs. T4: Treatment with 1% urea + 6.6% cottonseed meal and 5% rapeseed meal instead total soybean meal (19%) on Dumont lambs. * means *p* < 0.05, ** means *p* < 0.01, *** means *p* < 0.001.

Differences in “*Carbohydrate metabolism*”, “*Amino acid metabolism*”, and “*Energy metabolism*” among groups were analysed. Within “*Carbohydrate metabolism*” the most abundant pathways in order were *ko00520* (*amino sugar and nucleotide sugar metabolism*), *ko00500* (*starch and sucrose metabolism*), etc. *ko00052*, in the T4 group, was significantly lower than in T1 (*p* < 0.05), and the differences in enzymes within the pathway were analysed. It was found that the abundance of enzyme 2.7.1.58 in the T4 group was significantly lower than in T1 (*p* < 0.05), and the abundances of enzymes 2.4.1.82 and 3.2.1.26 in the T4 group were significantly higher than in the T1 group (*p* < 0.05). In the *amino acid metabolism*, it was found that *ko00220* (*arginine biosynthesis*) in T4 was significantly lower than that in the T1 and T3 groups (*p* < 0.05). In the T4 group, *arginine biosynthesis* was affected by the down-regulation of 1.4.1.2 and 3.5.1.54 and up-regulation of 3,5,3,6. In *the energy metabolism*, *ko00920* (*sulphur metabolism*) in the T4 group was significantly higher, and ko00710 (*carbon fixation in photosynthetic organisms*) was significantly lower than in the other groups (*p* < 0.05). The difference between groups was tested, and it was found that the enzymes with significant differences played a role. Compared with the T1 group, enzyme 1.8.4.8 was significantly decreased in the T4 group, while enzymes 1.8.5.3 and 3.1.3.7 were significantly increased in the T2 group (*p* < 0.05) (Figure A3).

**Figure 4:**
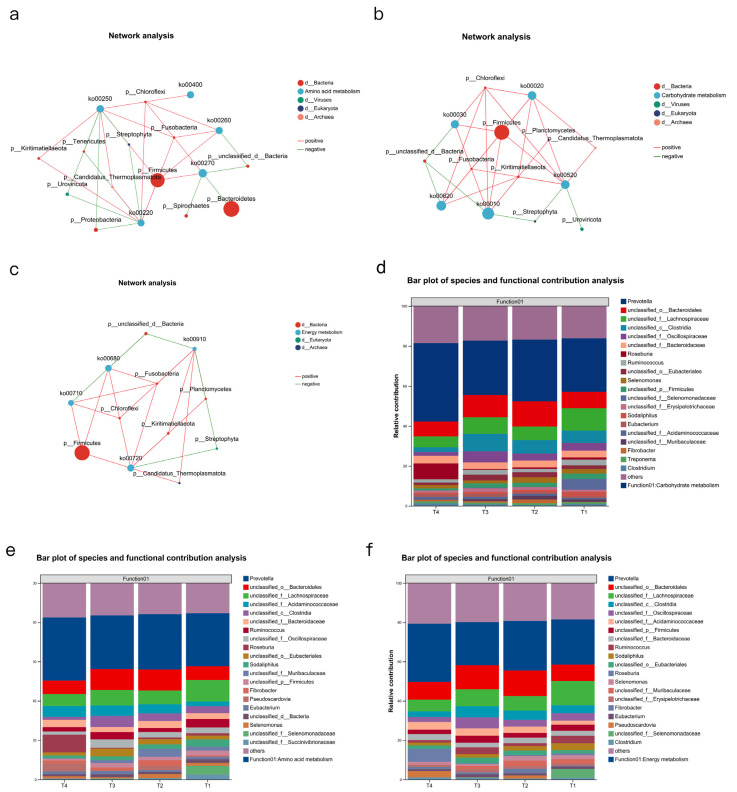
(**a**–**c**) Diagram illustrating the correlation network between bacteria phylum and microbial KEGG functions. (**a**) Amino acid metabolism. (**b**) Carbohydrate metabolism. (**c**) Energy metabolism. (**d**–**f**) Analysis of the contribution of bacterial genus to carbohydrate metabolism, amino acid metabolism, and energy metabolism. (**d**) Carbohydrate metabolism. (**e**) Amino acid metabolism. (**f**) Energy metabolism. T1: control group. T2: treatment with 1.5% urea as a replacement for 6.4% soybean meal in the diet of Dumont lambs. T3: treatment with 1% urea as a replacement for 4.3% soybean meal in the diet of Dumont lambs. T4: treatment with 1% urea +6.6% cottonseed meal and 5% rapeseed meal as a replacement for total soybean meal (19%) in the diet of Dumont lambs.

The rumen metabolic profile is also utilised for phenotypic (rumen fermentation) related analyses, where the top 10 most abundant metabolites are associated with rumen fermentation parameters for correlation analysis (Figure 4a–c). We conducted a comparative analysis of the types of microbiotas and these three differential pathways to explore how the microbiota affects the three major metabolic processes. It was found that in the three major metabolic processes, the microbiota groups T1, T2, and T3 had the highest contribution degrees, dominated by *Prevotella*, *unclassified_o__Bacteroidales*, and *unclassified_f__Lachnospiraceae*, respectively (Figure 4d–f). However, in group T4, the top three in contribution degree were *Prevotella*, *unclassified_o__Bacteroidales*, and *Roseburia*. Compared with the control group, the contribution degree proportion of *unclassified_f__Lachnospiraceae* in each test group decreased.

### 3.5. Rumen Metabolome

**Figure 5:**
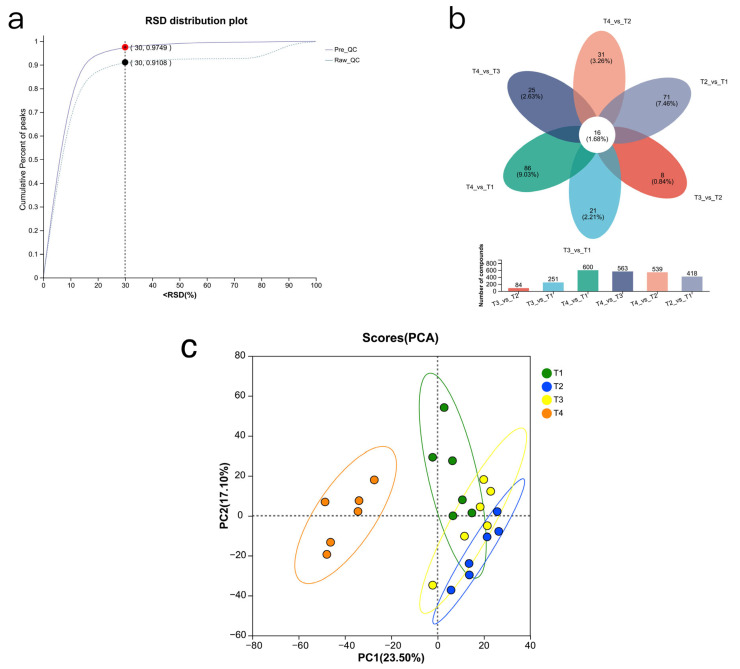
Rumen metabolome analysis. (**a**) RSD distribution plot (RSD < 30%, cumulative proportion of the ionic peaks > 0.7). (**b**) Venn plot. (**c**) PCA scores plot. T1: control group. T2: treatment with 1.5% urea as a replacement for 6.4% soybean meal in the diet of Dumont lambs. T3: treatment with 1% urea as a replacement for 4.3% soybean meal in the diet of Dumont lambs. T4: treatment with 1% urea + 6.6% cottonseed meal and 5% rapeseed meal as a replacement for total soybean meal (19%) in the diet of Dumont lambs.

A total of 1677 compounds were identified in the rumen metabolome, and there were 516 kinds of anionic metabolites (Figure 5a). The metabolic set was established, and Venn (Figure 5b) and PCA analyses (Figure 5c) were performed to observe the common and unique metabolites and the distance between metabolites. The results showed that the metabolite profile of the T4 group differed markedly from those in the other groups.

**Figure 6:**
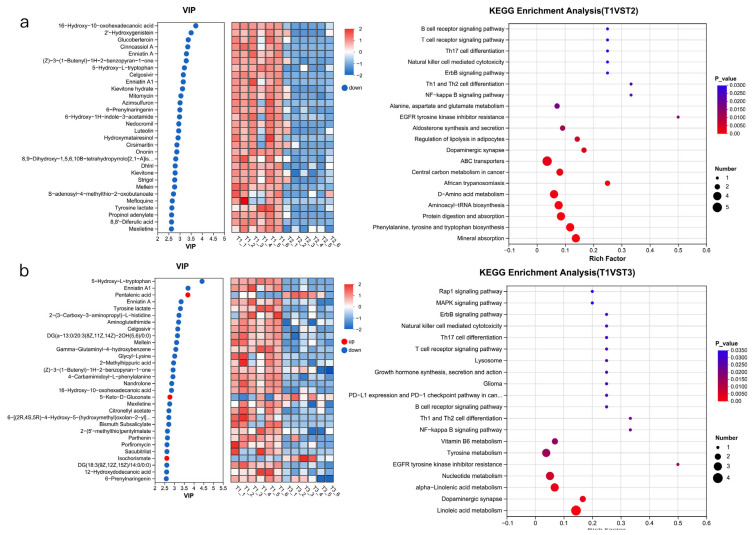

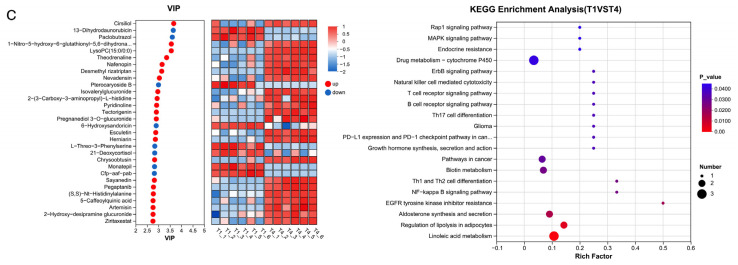
VIP analysis and KEGG enrichment analysis of the four groups. (**a**) VIP analysis and KEGG enrichment analysis in the T2 vs. T1 group comparison. (**b**) VIP analysis and KEGG enrichment analysis in the T3 vs. T1 group comparison. (**c**) VIP analysis and KEGG enrichment analysis in the T4 vs. T1 group comparison. T1: control group. T2: treatment with 1.5% urea as a replacement for 6.4% soybean meal in the diet of Dumont lambs. T3: treatment with 1% urea as a replacement for 4.3% soybean meal in the diet of Dumont lambs. T4: treatment with 1% urea + 6.6% cottonseed meal and 5% rapeseed meal as a replacement for total soybean meal (19%) in the diet of Dumont lambs.

Differential metabolite analysis was performed on the total metabolic set. After performing the Kruskal–Wallis H test and variable importance in projection (VIP) filtering on the relative concentrations of rumen metabolites, significant differences were observed between the test groups and control groups. Specifically, 134 differential metabolites were identified between T1 and T2 (Figure 6a), 88 differential metabolites were identified between T1 and T3 (Figure 6b), and 196 differential metabolites were identified between T1 and T4 (*p* < 0.05, VIP_PLS_DA > 2) (Figure 6c).

KEGG functional enrichment of important metabolites was performed. It was found that compared with T1, different metabolites were mainly enriched in *ABC transporters*, *D-amino acid metabolism*, *phenylalanine*, *tyrosine and tryptophan biosynthesis*, *protein digestion and absorption*, and *aminoacyl-tRNA biosynthesis* in the T2 group (Figure 6a). Compared with the control group, the differential metabolites in T3 were mainly enriched in *Aminoacyl-tRNA biosynthesis*, *alpha-Linolenic acid metabolism*, *nucleotide metabolism*, *nucleotide metabolism*, and *tyrosine metabolism* (Figure 6b). Compared with the control group, the different metabolites in T4 were mainly enriched in *drug metabolism-cytochrome P450* and *linoleic acid metabolism* (Figure 6c).

**Figure 7:**
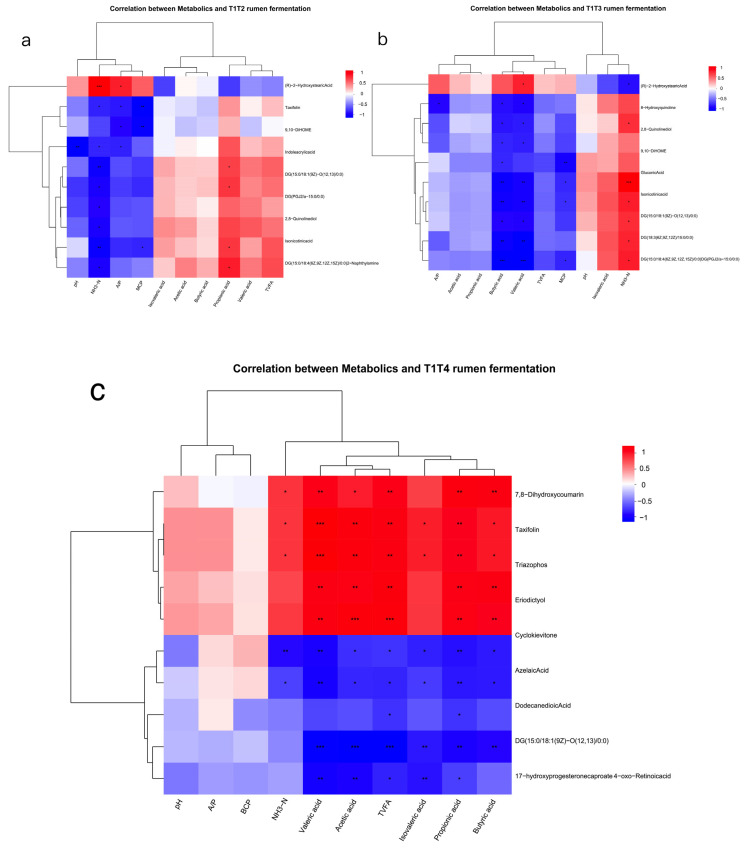
Correlation analysis between rumen metabolites and rumen fermentation parameters. (**a**) Correlation heatmap in T2 vs. T1. (**b**) Correlation heatmap in T3 vs. T1. (**c**) Correlation heatmap in T4 vs. T1. * means *p* < 0.05, ** means *p* < 0.01, *** means *p* < 0.001. T1: control group. T2: treatment with 1.5% urea as replacement for 6.4% soybean meal in the diet of Dumont lambs. T3: treatment with 1% urea as replacement for 4.3% soybean meal in the diet of Dumont lambs. T4: treatment with 1% urea + 6.6% cottonseed meal and 5% rapeseed meal as replacement for total soybean meal (19%) in the diet of Dumont lambs.

After correlating the rumen metabolites with rumen fermentation parameters, the metabolites with the same expression patterns were clustered. The rumen metabolic profile was also utilised for phenotypic (rumen fermentation)-related analyses, where the top 10 most abundant metabolites were associated with rumen fermentation parameters for correlation analysis (Figure 7a–c).

### 3.6. Rumen Fermentation Parameters, Rumen Microbial Species, and Metabolomics Association Analysis

**Figure 8:**
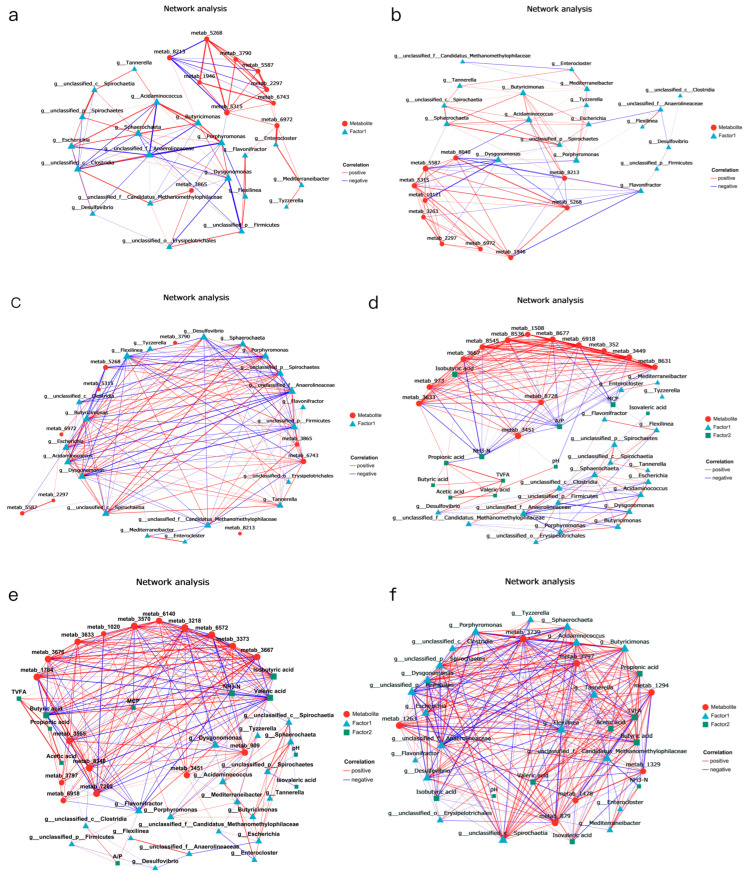
Correlation analysis of rumen fermentation, core rumen metabolites, and core rumen microbes. (**a**–**c**) Association analysis of the top microbial genus and metabolites related to phenotypic data. (**d**–**f**) Correlation analysis of experimental groups vs. T1. T1: control group. T2: treatment with 1.5% urea as a replacement for 6.4% soybean meal in the diet of Dumont lambs. T3: treatment with 1% urea as a replacement for 4.3% soybean meal in the diet of Dumont lambs. T4: treatment with 1% urea + 6.6% cottonseed meal and 5% rapeseed meal as a replacement for total soybean meal (19%) in the diet of Dumont lambs.

We conducted a correlation analysis between the top metabolites related to phenotypic data and the top rumen genera. It was found that in group T2, the genus *Mediterraneibacter* was significantly positively correlated with 9,10-DiHOME (*p* < 0.05), and the genus *Butyricimonas* was significantly positively correlated with (R)-2-Hydroxystearic acid (*p* < 0.05). Additionally, *unclassified f Candidatus Methanomethylophilaceae* and *unclassified o Erysipelotrichales* were significantly positively correlated with 2-Naphthylamine (*p* < 0.05) (Figure 8a). In group T3, the genera *Acidaminococcus* and *Butyricimonas* were significantly positively correlated with (R)-2-Hydroxystearic acid (*p* < 0.01) (Figure 8b). In group T4, *unclassified p Firmicutes* was positively correlated with 2-Naphthylamine, and *Acidaminococcus* was positively correlated with taxifolin (*p* < 0.05) (Figure 8c).

The rumen metabolome was also used for microbial association analysis and to identify significant differences. Therefore, we used the top 10 most abundant related metabolites and the top 10 most abundant rumen microorganisms for correlation heat map analysis (Figure 8d–f). When combined with rumen microorganism in T1 vs. T2, we found that four metabolites (8, 9-dihydroxy-1,5,6,10B-tetrahydropyrrolo [2, 1-a] isoquinolin-3(2H)-one, mitomycin, and mexiletine) could be better correlated with two microbial genera (*g__Enterocloster* and *g__unclassified_f__Anaerolineaceae*) (Figure 8d).

A relationship between metabolites and rumen microbial genera also exists in the T1 vs. T3 comparison, where we identified seven metabolites (Isochorismate, 16-Hydroxy-10-oxohexadecanoic acid, DG(a-13:0/20:3(8Z,11Z,14Z)-2OH(5,6)/0:0), Gamma-Glutaminyl-4-hydroxybenzene, 12-Hydroxydodecanoic acid, mexiletine, and citronellyl acetate) that could be better correlated with six genera (Figure 8e).

Some bacteria in group T4 showed a significant positive correlation with some metabolites and a negative correlation with others. For example, *G__fovibrio*, *g__Acidaminococcus*, *g__Butyricimonas*, *g__Sphaerochaeta*, *g__unclassified_c__Spirochaetia* were significantly correlated with metabolites such as (S, S)-Nt-Histidiny lalanine, 2-(3-Carboxy-3-aminopropyl) -L-Histidine, and 2-hydroxy-Desipramine glucuronide, while showing significant negative correlations with paclobutrazol (Figure 8f).

A multi-factor correlation network diagram was constructed using rumen fermentation phenotypic data, the top 10 most abundant differential strains, and the top 30 VIP-ranked metabolites. The size of the nodes was used to determine whether the three characteristic data were closely related, and whether rumen bacteria affected rumen fermentation by influencing the metabolites.

## 4. Discussion

### 4.1. Growth Performance and Rumen Fermentation Characteristics

Body weight (BW), average daily weight gain (ADG), and average daily feed intake (ADFI) are important indicators reflecting the growth performance of livestock, which can directly reflect the growth status of the animal’s body. In this experiment, replacing part of the soybean meal with urea, cottonseed meal, and rapeseed meal did not change the BW, ADG, and ADFI of Dumont lambs, indicating that appropriate dosages of urea, cottonseed meal, and rapeseed meal do not have any negative impact on the lambs when used in conjunction with cost reduction measures. Studies [19] have shown that in the research on lambs, using 10 g urea to replace 130 g/kg DM soybean meal has no adverse effect on the digestion, metabolism, and growth of low-protein and high-concentrate-fed fattening Hu sheep, but as the urea replacement level increases, the growth performance of Hu sheep decreases. Similarly, replacing 6.4% soybean meal with 1.5% urea in the lamb diet has no significant effect on the DM intake and digestion coefficient of the animal, but increasing the replacement level to 2.5% will reduce the DM and organic matter intake of the animal [20]. Feed conversion ratio (FCR) is a key indicator for evaluating the economic benefits of sheep farming and directly affects feed utilization efficiency. In this experiment, replacing all soybean meal with 1% urea, cottonseed meal, and rapeseed meal significantly increased the FCR of Dumont lambs, a result that is conducive to improving the economic benefits of farming and achieving economic maximization.

The rumen is a natural anaerobic space that provides the essential environment for the rumen microbiota to reproduce and develop. During the fermentation process, the rumen microbiota synthesizes substances such as VFA and NH_3_-N, which are important indicators of the physiological health of the rumen and the ability of ruminants to convert nutrients. Rumen pH is an important indicator for evaluating rumen fermentation status, which is jointly affected by multiple factors [21]. In this experiment, the pH values of each group were not significantly different, which was consistent with the results of [6,22]. When there was an adequate amount of fermentable carbohydrates in the diet, the rate of NH_3_-N release matched the microbial requirements.

Ammonia is an important source of nitrogen for MCP synthesis and growth in the rumen, and the rumen microorganisms can utilise the NH_3_-N produced by degradation as a nitrogen source. Through amino acid deamination or NPN hydrolysis, ammonia is generated, and then ammonia is converted into microbial protein. The synthesized and transformed MCP can provide 50% to 80% of the absorbable protein for ruminant animals [23]. Urea, which can be rapidly hydrolysed to ammonia by rumen ureolytic microbes, leads to an increase in the concentration of NH_3_-N [24]. When the energy–nitrogen synchronization degree in the rumen is high, microorganisms can more effectively utilise the nitrogen source, reduce ammonia loss, and promote microbial protein synthesis [25]. In this experiment, different doses of urea were used to replace part of the soybean meal, and the NH_3_-N content was significantly different, but the contents of MCP were significantly increased. This indicates that replacing 4.3% soybean meal with 1% urea improved the utilization efficiency of CP in the diet by rumen microorganisms, which is related to the growth of protein-degrading microorganisms in the rumen. Consistent with the research results of these individuals [26], they also reported that 1.5% urea would increase the NH_3_-N value, and 1% urea could enhance the production of MCP.

VFA is the main source of energy in ruminants, accounting for 70% to 80% of digestible energy intake in ruminants, and is the main energy source of ruminants [27]. In this experiment, acetic acid content in each group accounted for the most TVFA, indicating that acetic acid was the main factor in the rumen fermentation of sheep fed different nitrogen sources. Compared with other groups, acetic acid and propionic acid contents in the T4 group were significantly increased. This is related to the fact that cottonseed meal contains more crude protein [28], while rapeseed meal has a higher cellulose content. This will increase the number of fibrolytic bacteria and anaerobic fungi in the rumen and promote acetic acid fermentation in the rumen. Isobutyric acid serves as an energy source for ruminants and is an important metabolic product of cellulolytic bacteria in the rumen, such as succinic acid, fibrobacter, and rumen bacteria. The changes in its content are closely related to the activity of these microorganisms [29]. In this experiment, the isobutyric acid content in the T3 group was significantly lower than that in the other groups. This suggests that the decrease in the pH value might have inhibited or reduced the activity of the cellulose-degrading bacteria [30]. Studies have shown that an increase in the non-fibre carbohydrate (NFC) level of the diet leads to a decrease in the rumen ethyl-pentyl ratio. In high-concentrate diets, the NFC level is high, and the oligosaccharides and monosaccharides produced by NFC degradation can only be utilised by a portion of the microorganisms. In high-concentrate diets, many microorganisms related to NFC degradation multiply in the rumen. The single sugars or oligosaccharides produced by NFC degradation are quickly captured and utilised by these types of microorganisms, and these microorganisms are often closely related to the production of propionic acid, inlcuding starch-degrading bacteria and lactic acid-producing bacteria [31].

### 4.2. Rumen Microbial Characteristics

The rumen microbiota of ruminant animals is an important component of their digestive system. In this study, the urea, cottonseed meal, and rapeseed meal contained in the T4 group were rich in sulphur-containing amino acids, providing a more abundant nitrogen source for the T4 rumen microbiota, thereby enhancing microbial diversity, consistent with the findings of [32]. Our data indicate that the rumen of Dumont lambs is dominated by the *Bacteroidetes* and *Firmicutes* phyla regardless of diet, with the abundance of *Firmicutes* ranking second only to *Bacteroidetes* at the phylum level. Firmicutes utilise free enzymes and cellulosomes for polysaccharide degradation, while Bacteroidetes employ polysaccharide utilization loci [33]. They can convert plant fibres into low-molecular-weight carbohydrates and short-chain fatty acids (mainly butyric acid), providing energy sources for rumen microorganisms and nutrients for host animals [25].

At the genus level, *Prevotella* are key players in hemicellulose digestion and contribute significantly to the degradation of starch, cellulose, and pectin in the rumen [34]. Their ability to produce propionate as a fermentation product may help reduce methane emissions in ruminants [35]. Our study showed that in the T4 group, *Prevotella* abundance increased but there was no significant difference. An increase in rumen *Prevotella* has been linked to enhanced branched-chain amino acid synthesis, potentially contributing to improved muscle growth in ruminants [36].

We also found that the microbial communities of certain genera underwent significant changes across the four treatment groups. In the urea-added treatment group, the abundance of *Acidaminococcus* significantly increased. Research indicates that bacteria belonging to the *Acidaminococcus* genus can grow using amino acids as their sole energy source and produce volatile fatty acids (VFAs) [37,38]. These bacteria produce metabolic products such as acetic acid and butyric acid through amino acid metabolism, thereby influencing the balance of the rumen microbial community and the host’s absorption of nutrients [37]. The abundance of *Acidaminococcus* is positively correlated with the total volatile fatty acid (TVFA) concentration in the rumen. Research indicates that the association between dominant rumen bacterial communities and methanogenic archaea is relatively weak. However, certain bacteria with comparatively low abundance exhibit strong correlations with methanogenic archaea, such as the succinate-producing Succinivibrionaceae, the succinate-utilising Dialister, and the amino-acid-fermenting Acidaminococcus, as well as methanogens belonging to the *Methanomassiliicoccaceae*, *Methanosphaera* sp. A4, and *Methanobrevibacter boviskoreani*. *Succinivibrio* spp. [39]. Replacing part of the soybean meal with urea can effectively regulate the rumen microbial community and enhance fermentation efficiency. However, this is accompanied by a slight increase in methane emissions, and the T2 group also showed a significant increase in the abundance of *Candiatus_Themoplasmatota*, accompanied by increased methane production [40].

The *Sphaerochaeta* genus is an important component of the rumen microbial community, and its metabolic characteristics are mainly reflected in fermentation and carbohydrate metabolism The genomes are highly enriched in fermentation and carbohydrate metabolism genes, many acquired from non-spirochetes, particularly *clostridia* [41]. Sphaerochaeta genus exhibit pleomorphic cell shapes including spherical, annular, curved rod, helical, and coccoid forms [42]. They are chemoorganotrophic fermenters that utilise various carbohydrates, producing acetate, ethanol, hydrogen, and carbon dioxide as major end products, acetic acid supplies 70% of the body’s energy requirements and is utilised in the synthesis of body fat and milk fat. The hydrogen it produces is utilised by methanogenic bacteria to reduce CO_2_ into methane. However, in the presence of sulphate-reducing bacteria or nitrate-reducing bacteria, hydrogen is employed to reduce sulphate or nitrate. This process competes with methanogenesis and can effectively reduce methane emissions [41,42]. Research has found that the *globotrichonium* genus can generate acetic acid, lactic acid, and ethanol in the rumen using galacturonic acid and pectin, and these products are key intermediates in rumen methane production [43]. Additionally, this bacterium is positively correlated with rumen nitrogen content [44], which may also be one of the reasons for the significantly higher ammonia nitrogen content observed in the T4 group.

### 4.3. Rumen Microbial Functions

According to the KEGG function, we found that all groups had abundant “amino acid metabolism”, “carbohydrate metabolism” and “energy metabolism” functions regardless of diet. Interestingly, although all used urea, the results of its KEGG function enrichment at level 3 were different. The T2 group exhibited significantly abundant fructose and mannose metabolism ability, indicating that the rumen microorganisms in this group can effectively utilise the intermediate products of fibre degradation and avoid carbon waste [45]. The ability of rumen microorganisms to utilise polysaccharides and succinic acid to produce acetic acid and propionic acid was enhanced, but the final VFA content was not significantly affected, while MCP was significantly increased. This indicates that replacing 6.4% the soybean meal with 1.5% urea is beneficial for the synthesis of polysaccharides, fumaric acid and other substances, and that the efficiency of microbial utilisation of VFA is enhanced [46].

The T3 group has abundant ABC transporters functions. In rumen bacteria, ABC transporters are involved in carbohydrate uptake. These transporters use ATP hydrolysis to drive substrate translocation across membranes [47]. Ruminal bacteria employ various transport mechanisms, including passive diffusion for hydrophobic substances and carrier-mediated transport for hydrophilic compounds. ABC transporters are membrane proteins that use ATP energy to transport various substances across biological membranes [48]. They play crucial roles in cellular physiology, including nutrient import, toxin export, and protection against xenobiotics [49]. This also explains the reason for the significant increase in microbial protein content in the T3 group. These proteins are also involved in mitochondrial membrane protein degradation, working alongside AAA proteases and other components to maintain membrane integrity and function [50]. Additionally, ABC transporters participate in peptide trafficking and translocation, which is essential for cellular signalling and self-defence mechanisms [51]. The peptides resulting from protein degradation can serve as signalling molecules or be further broken down for amino acid recycling and energy production [51]. Ribosomes play a crucial role in protein synthesis and cellular function. Ribosomes are essential for mRNA selection, protein translation, and folding, with quality control mechanisms ensuring proper assembly and functionality [52].

The T4 group has the richest ribosome, indicating that the rumen microorganisms are in an active proliferation state and require a large amount of synthetases (such as cellulase, amylase) and structural proteins. It is related to the host’s high feed utilization efficiency in the T4 group.

### 4.4. The Correlation Between Rumen Microbial Communities, Growth Performance, and Rumen Fermentation in Dumont Lambs

In this study, by replacing 4.3% the soybean meal with 1% urea, rapeseed meal, and cottonseed meal in the diet of Dumont lambs, it was found that the enrichment of arginine biosynthesis was significantly higher than in other groups, and there was a significant positive correlation between the *Firmicutes* phylum and *Chloroflexi* in the rumen and arginine biosynthesis. This finding is related to the regulation of microbial metabolism and amino acid metabolic pathways by changes in protein source components. Additionally, the addition of urea may promote microbial protein synthesis by providing a non-protein nitrogen (NPN) source, thereby influencing amino acid metabolic pathways. The increased arginine synthesis provides precursor substances for propionic acid production [53], which is also one of the reasons for the improved feed conversion efficiency in the T4 group. Consistent with study [54], this study found that cows with high feed conversion efficiency had higher propionic acid levels in the rumen. Additionally, study [55] indicated that increased arginine metabolism in the rumen is associated with enhanced microbial community activity, particularly among microorganisms involved in the urea cycle.

In the contribution analysis of amino acid metabolism and carbohydrate metabolism, regardless of diet, *Prevotella* plays a crucial role in the metabolism of carbohydrates, lipids, and amino acids in the rumen and is correlated with livestock growth performance [56]. *Prevotella*’s ability to produce propionate during fermentation may reduce methane emissions in ruminants [35]. Studies have shown a correlation between *Prevotella* abundance and improved glucose metabolism in both humans and animals [57]. The urea-added group reduced the abundance of *unclassified Lachnospiraceae*. Studies indicate that its isolates exhibit metabolic flexibility when exposed to different substrates and hydrogen concentrations, producing multiple end products, including acetate, butyrate, and hydrogen [58].

The 1% urea, rapeseed meal, and cottonseed meal group increased *Roseburia* abundance. *Roseburia* bacteria produce short-chain fatty acids, particularly butyrate, which play a crucial role in colon health, immune function, and anti-inflammatory properties [59]. In energy metabolism, the abundance of Clostridium increased, capable of fermenting cellulose and other carbohydrates [60]. However, their content in the rumen is extremely low, indicating limited contributions to rumen metabolism [61]. This suggests that dietary regulation can influence rumen fermentation, amino acid metabolism, carbohydrate metabolism, and energy metabolism by altering the structure and function of the microbiota, primarily through the regulation of the *Prevotella* genus.

### 4.5. Rumen Metabolites

Changing the nitrogen source in the feed can affect rumen metabolites by altering the diversity or relative abundance of rumen microorganisms [62].

In this study, Dumont lambs fed urea, rapeseed meal, and cottonseed meal had more unique metabolites. The dry matter (DM), organic matter (OM), and crude protein (CP) degradation rates and effective degradation rates of soybean meal in the rumen were higher than those of cottonseed meal and rapeseed meal, and the rumen retention time was shorter. This indicates that cottonseed meal and rapeseed meal degrade relatively slowly, prolonging microbial utilisation time and promoting the accumulation of various metabolic products. Additionally, replacing soybean meal with urea, cottonseed meal, and rapeseed meal simultaneously increased rumen volatile fatty acid (VFA) production and microbial diversity, resulting in the generation of more specific metabolites.

In the KEGG functional enrichment analysis, the functional enrichment in the T2 group primarily involved enhanced amino acid metabolism and synthesis, as well as protein digestion, absorption, and transport. This indicates that the use of rapidly degradable proteins facilitates the activation of nutrient digestion within the rumen and meets the microbial demand for protein synthesis.

In addition, the ammonia produced by urea decomposition can be used by rumen microorganisms for amino acid synthesis, further participating in the proliferation, apoptosis, absorption and transport functions of rumen epithelial cells. The synthesis of amino acids may promote the metabolism of D-amino acids in the rumen, enhance the biosynthesis of aminoacyl-tRNA, and regulate the expression and activity of functional enzymes and genes involved in phenylalanine, tyrosine, and tryptophan metabolism. These processes are important components of microbial proteins.

The 1% urea, rapeseed meal, cottonseed meal group had a significantly enriched “Linoleic acid metabolism” KEGG function. It indicated that cotton meal and rapeseed meal contain a high proportion of linoleic acid, and rumen microorganisms need to activate metabolic pathways to hydrogenate it into conjugated linoleic acid and stearic acid [32]. The enrichment of metabolic pathways indicates that the hydrogenation process is active. The hydrogenation process of linoleic acid consumes H_2_, which may reduce methane emissions, but also results in the loss of some dietary energy [63]. Studies have shown that rapid hydrolysis of urea leads to a short-term spike in rumen ammonia concentration, inhibiting some fibre-degrading bacteria such as *Ruminococcus flavefaciens*, while ammonia-tolerant bacteria such as *Prevotella* proliferate relatively more, endowing them with stronger lipid metabolism ability [64]. In addition, some rumen microorganisms (such as *Clostridium*) can promote nitrogen assimilation through short-chain fatty acids (such as propionic acid) produced by linoleic acid metabolism, helping to maintain ammonia homeostasis [65]. In the KEGG functional enrichment analysis of other groups, we found that the T2 group exhibited abundant ABC transporters functions, etc.

### 4.6. Relation Analysis

Urea is a type of rapidly degradable protein, various bacterial species have been identified as ureolytic, including *Selenomonas ruminantium*, *Lactobacillus* sp., *Enterococcus* sp., and *Staphylococcus* sp. [66]. Other ureolytic species include *Peptostreptococcus productus*, *Ruminococcus albus*, and *Succinivibrio dextrinosolvens* [67]. Recent studies using high-throughput sequencing have identified additional ureolytic genera, such as *Pseudomonas*, *Haemophilus*, *Neisseria*, and *Bacillus* [68]. Rumen microorganisms participate in urea hydrolysis through compartment—specific activities. Several studies report that bacteria adhering to the rumen wall display the highest urease activity—with over 55% of ureC gene sequences remaining unclassified—indicating a reservoir of novel taxa [69]. Liquid-associated bacteria (e.g., *Prevotella* and *Succinivibrionaceae*) exhibit intermediate enzyme activity, while solid-associated microbes (including fibre degraders such as *Lachnospiraceae*) contribute at lower, yet functionally significant, levels [54].

In this experiment, when 1.5% urea was used to replace 6.4% soybean meal, the rumen microbiota dominated by *Enterocloster* influenced the accumulation of rumen fermentation-related metabolites such as 9,10-DiHOME, DG (PGJ2/a-15:0/0:0), and isonicotinic acid. Urea is rapidly hydrolysed by urease in the rumen into ammonia (NH_3_-N), leading to an increase in NH_3_-N concentration in the rumen. Excessive NH_3_-N promotes the proliferation of bacteria such as *Enterocloster*, thereby affecting the synthesis of the aforementioned metabolites.

When 1% urea replaced 4.3% the soybean meal, the microbial community, dominated by *Acidominococcus*, affected the accumulation of DG(PGJ2/a-15:0/0:0), and this metabolite showed a significant positive correlation with NH_3_-N content and a negative correlation with MCP. This phenomenon is related to changes in the fermentation rate of carbohydrates in the rumen after replacing part of the soybean meal with urea [70]. *Acidaminococcus* has been shown to utilise citric acid and trans-citric acid as energy sources, producing hydrogen and hydrogen sulphide. The high NH_3_-N concentration generated by urea degradation inhibits *Acidominococcus* activity by competing with hydrogen sulphide for H2, thereby enhancing the rumen microbial conversion efficiency of NH_3_-N and increasing the MCP content. When 1% urea meal and cottonseed meal replaced soybean meal, the accumulation of *Sphaerochaeta* and taxifolin showed a positive correlation, and taxifolin was also extremely significantly positively correlated with TVFA and VFA components. *Taxifolin*, a flavonoid compound with potent antioxidant properties, has shown promising therapeutic potential across various biological systems. It protects retinal pigment epithelial cells against oxidative stress-induced apoptosis by activating NRF2 and enhancing phase II antioxidant enzymes [71]. While not directly related to rumen VFA, taxifolin has been shown to alter gut microbiota and increase short-chain fatty acid production, particularly butyric acid, in mice with induced colitis [72].

Consistent with this study, many studies have shown that [20,73] certain metabolites are positively correlated with the formation of specific bacterial groups, while negatively correlated with the formation of other bacterial groups, and this is related to different feed utilization efficiencies. The relationships between the rumen microbial taxa, functions, and metabolome provide new insights into the functional roles of the rumen microbiome in producing small molecule metabolites and contributing to host traits. Also, this article has certain limitations. After replacing part of the soybean meal with urea, rapeseed meal, and cottonseed meal in the diet of Dumont lambs, the research mainly focused on the rumen part. In the future, based on an expanded sample size, this study should be extended to the hindgut to clarify how urea, cottonseed meal, and rapeseed meal affect the absorption, digestion, and metabolism of nutrients in the host. The specific mechanism still needs further research.

## 5. Conclusions

The degradation rates of urea, rapeseed meal, and cottonseed meal in the rumen differ, stimulating carbon metabolism and arginine synthesis pathways primarily mediated by *Precolleta*, thereby affecting the accumulation of metabolites such as 9,10-DiHOME, DG (PGJ2/a-15:0/0:0), isonicotinic acid, and taxifolin, which in turn influence rumen fermentation. Replacing part of the soybean meal with urea in the diet of Dumont lambs can increase microbial protein (MCP) production, which aids in the absorption of subsequent nutrients. Replacing all soybean meal with 1% urea + 5% rapeseed meal + 6.6% cottonseed meal can improve rumen fermentation, enhance rumen microbial and metabolite diversity, and optimise the synergistic metabolic efficiency of carbon, nitrogen, and sulphur. However, the specific mechanisms of post-rumen digestion and metabolism require further investigation.

## Figures and Tables

**Table 1:** Experimental diet composition and nutrient level (air-dried basis, %).

Items	Groups			
	T1	T2	T3	T4
Corn	43.00	50.00	49.00	49.00
Soybean meal	19.00	8.00	12.00	0.00
Cottonseed meal	-	-	-	6.60
Rapeseed meal	-	-	-	5.00
Wheat bran	3.70	-	-	0.00
Distillers Dried Grains with Solubles	-	7.05	4.60	6.00
Sunflower peel	20.00	20.00	20.00	19.10
Peanut shell powder	11.00	10.00	10.00	10.00
Urea ^2^	0.00	1.50	1.00	1.00
Nacl	0.50	0.50	0.50	0.50
NaHCO_3_	1.00	1.00	1.00	1.00
Premix ^1^	1.00	1.00	1.00	1.00
CaSO_4_	0.80	0.80	0.80	0.80
CaHCO_3_	-	0.15	0.10	-
Total	100.00	100.00	100.00	100.00
Nutrient levels ^3^				
DE/(MJ/kg) ^2^	11.12	11.06	11.13	11.08
DM	88.70	88.65	88.64	88.54
CP	14.05	14.02	14.06	14.02
EE	2.29	2.89	2.68	2.99
NDF	30.08	29.94	29.45	30.77
ADF	21.83	20.80	20.85	21.60
ADL	3.27	3.15	3.17	3.25
OM	85.73	86.13	86.02	86.04
Ca	0.69	0.71	0.73	0.69
P	0.34	0.31	0.32	0.35
Thr	0.57	0.49	0.52	0.55
Met	0.20	0.18	0.19	0.20
Lys	0.67	0.42	0.50	0.40
Arg	0.85	0.61	0.72	0.84
TAA	11.62	10.95	11.04	11.10

^1^ The premix provides each kilogram of feed with the following: Ca 1520.00 mg, P 410.00 mg, Fe 25.00 mg, I 0.90 mg, Zn 35.00 mg, Co 0.10 mg, Co 9.00 mg, Se 0.25 mg, Mn 19.50 mg, vitamin E 15 IU, vitamin D_3_ 1000.00 IU, vitamin A 3000.00 IU, and vitamin B_3_ 60.00 mg. ^2^ The urea used is a premixed form with a protein content of 200%, in accordance with the “Feed Additive Safe Use Specifications” issued by the Ministry of Agriculture of the People’s Republic of China—No. 2625. ^3^ The nutritional levels are calculated values, while the rest are measured values. DE: digestible energy; DM: dry matter; CP: crude protein; EE: ether extract; NDF: neutral detergent fibre; ADF: acid detergent fibre; ADL: acid detergent lignin; OM: organic matter; Ca: calcium; P: phosphorus; Thr: threonine; Met: methionine; Lys: lysine; Arg: arginine; TAA: total amino acid.

**Table 2:** Effects of urea, cottonseed meal, and rapeseed meal as partial replacements for soybean meal on growth performance of Dumont lambs.

Items ^1^	T1 ^2^	T2 ^2^	T3 ^2^	T4 ^2^	SEM ^3^	*p*-Value ^4^
BW (kg)						
0 d	23.56	23.51	23.08	22.98	0.87	0.91
30 d	28.51	29.11	28.78	30.00	1.30	0.67
60 d	35.19	36.75	35.21	37.10	1.49	0.45
90 d	40.70	39.46	41.30	42.38	1.99	0.53
ADG (g/d)						
0–30 d	195.70	193.10	198.30	208.60	31.19	0.96
30–60 d	228.70	243.30	210.80	245.80	27.52	0.60
60–90 d	232.50	225.70	240.00	227.30	21.80	0.96
0–90 d	217.50	212.40	231.50	241.20	22.94	0.60
ADFI (g/d)						
0–30 d	1194.00	1223.00	1214.00	1233.00	36.46	0.74
30–60 d	1501.00	1512.00	1503.00	1512.00	7.75	0.40
60–90 d	1682.00	1737.00	1718.00	1745.00	28.70	0.18
0–90 d	1407.00	1436.00	1424.00	1442.00	18.33	0.30
FCR						
0–90 d	7.48 AB	8.12 A	7.99 A	6.23 B	0.46	0.01

^1^ BW: body weight. ADG: average daily gain. ADFI: average daily feed intake. FCR: feed conversion ratio. ^2^ T1: control group. T2: treatment with 1.5% urea as a replacement for 6.4% soybean meal in the diet of Dumont lambs. T3: treatment with 1% urea as a replacement for 4.3% soybean meal in the diet of Dumont lambs. T4: treatment with 1% urea + 6.6% cottonseed meal and 5% rapeseed meal as a replacement for total soybean meal (19%) in the diet of Dumont lambs. ^3^ SEM = standard error of the mean. ^4^ ANOVA = contrast between T1, T2, T3, and T4. Different letters above the bars of the same row indicate significant differences (*p* < 0.05), while the same letters or no letters indicate no significant differences (*p* > 0.05).

**Table 3:** Effects of urea, cottonseed meal, and rapeseed meal as replacements for soybean meal on the rumen fermentation of Dumont lambs.

Items	T1 ^2^	T2 ^2^	T3 ^2^	T4 ^2^	SEM ^3^	*p*-Value ^4^
pH	6.63	6.75	6.71	6.74	0.03	0.60
NH_3_-N (mg/100 mL)	16.69 B	17.19 A	16.64 B	16.84 B	0.04	*p* < 0.01
TVFA ^1^ (mmol/L)	62.66 C	67.17 C	79.12 B	97.27 A	2.71	*p* < 0.01
VFA (mmol/100 mmol)						
Acetic acid	52.09 A	52.71 A	47.25 B	48.17 AB	1.94	*p* < 0.01
Propionic acid	39.56	39.28	39.03	40.80	0.52	0.79
Butyric acid	4.13 B	4.63 B	9.08 A	7.77 A	0.21	*p* < 0.01
Isobutyric acid	1.07 A	0.70 B	0.61 B	0.53 B	0.06	*p* < 0.01
Isovaleric acid	2.02 A	1.86 AB	1.85 AB	1.67 B	0.04	0.02
Valeric acid	2.36 B	1.94 C	3.30 A	2.23 BC	0.02	*p* < 0.01
A/P	1.37	1.34	1.21	1.19	0.03	0.40
MCP ^1^ (g/L)	4.06 C	4.11 A	4.09 AB	4.09 B	0.01	*p* < 0.01

^1^ TVFA: total volatile fatty acid. MCP: microbial protein. ^2^ T1: control group. T2: treatment with 1.5% urea as a replacement for 6.4% soybean meal in the diet of Dumont lambs. T3: treatment with 1% urea as a replacement for 4.3% soybean meal in the diet of Dumont lambs. T4: treatment with 1% urea + 6.6% cottonseed meal and 5% rapeseed meal as a replacement for total soybean meal (19%) in the diet of Dumont lambs. ^3^ SEM = standard error of the mean. ^4^ ANOVA = contrast between T1, T2, T3, and T4. Different letters above the bars of the same row indicate a highly significant difference (*p* < 0.01), while the same letters or no letters indicate no significant differences (*p* > 0.05).

**Figure A1:**
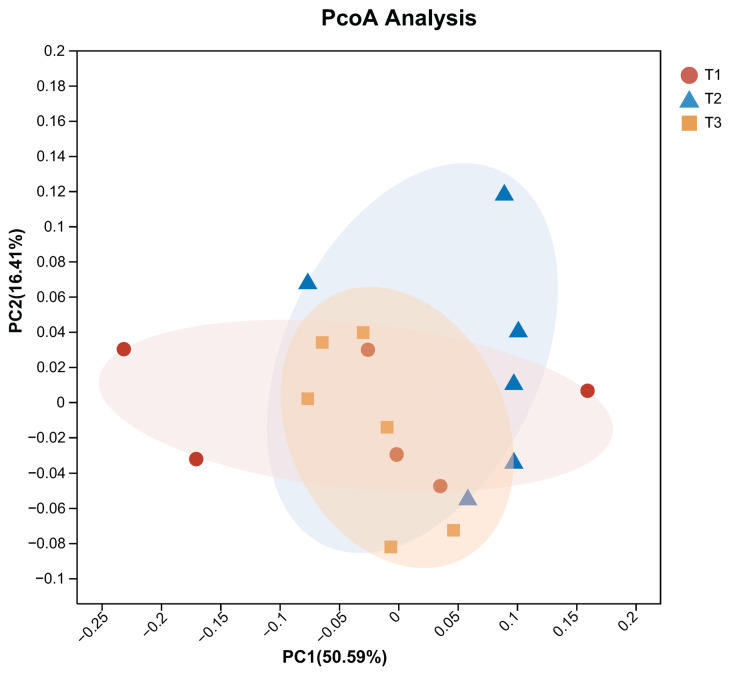

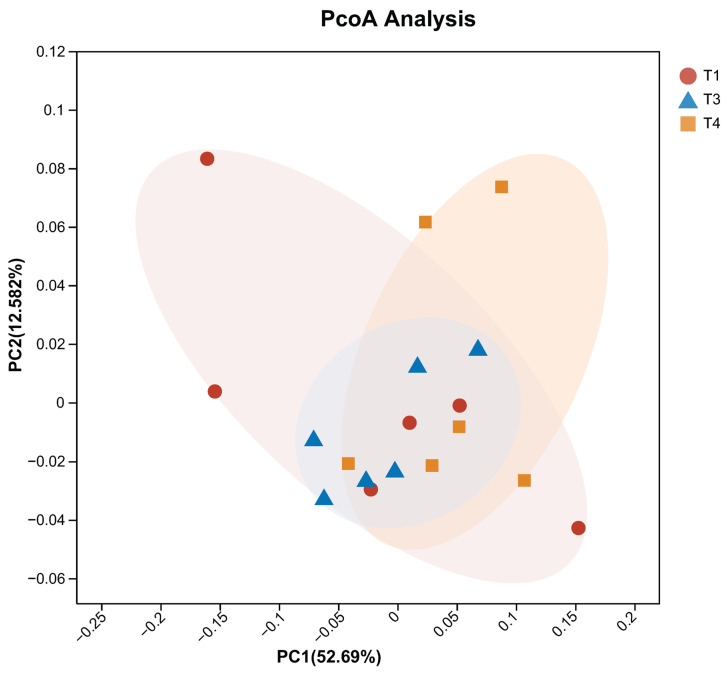
PcoA analysis of the T1, T2, T3 groups and T1, T3, T4 groups. T1: Control group. T2: Treatment with 1.5% urea instead 6.4% soybean meal on Dumont lambs. T3: Treatment with 1% urea instead 4.3% soybean meal on Dumont lambs. T4: Treatment with 1% urea + 6.6% cottonseed meal and 5% rapeseed meal instead total soybean meal (19%) on Dumont lambs.

## Data Availability

The datasets used in this study are available from the corresponding author on reasonable request.

## Abbreviations

The following abbreviations are used in this manuscript:

MCP	Microbial Proteins
NPN	Non-protein Nitrogen
DMI	Dry matter intake
ADG	Stage Daily gain
FCR	Feed conversion ratio
VFA	Volatile fatty acid
TVFA	Total volatile fatty acid
NFC	Non-fiber carbohydrate

## Appendix A

**Table A1:** Original sequence statistics table.

Samples	Insert Size (bp)	Read Length (bp)	Raw Reads	Raw Base (bp)
T1_1	500	150	43,525,650	6,572,373,150
T1_2	500	150	46,255,592	6,984,594,392
T1_3	500	150	47,285,324	7,140,083,924
T1_4	500	150	51,987,214	7,850,069,314
T1_5	500	150	47,107,196	7,113,186,596
T1_6	500	150	43,809,074	6,615,170,174
T2_1	500	150	41,042,824	6,197,466,424
T2_2	500	150	46,652,884	7,044,585,484
T2_3	500	150	46,157,166	6,969,732,066
T2_4	500	150	43,980,538	6,641,061,238
T2_5	500	150	41,223,064	6,224,682,664
T2_6	500	150	42,362,916	6,396,800,316
T3_1	500	150	48,975,558	7,395,309,258
T3_2	500	150	55,909,338	8,442,310,038
T3_3	500	150	49,054,578	7,407,241,278
T3_4	500	150	49,301,828	7,444,576,028
T3_5	500	150	47,961,358	7,242,165,058
T3_6	500	150	45,708,672	6,902,009,472
T4_1	500	150	44,642,316	6,740,989,716
T4_2	500	150	50,785,776	7,668,652,176
T4_3	500	150	52,399,324	7,912,297,924
T4_4	500	150	46,906,130	7,082,825,630
T4_5	500	150	46,731,708	7,056,487,908
T4_6	500	150	46,156,316	6,969,603,716

**Table A2:** Quality control sequence statistics table.

Samples	Clean Reads	Clean Base (bp)	Percent in Raw Reads (%)	Percent in Raw Bases (%)
T1_1	42,421,530	6,396,426,098	97.463288888276	97.322929663542
T1_2	45,189,544	6,813,348,801	97.695310007058	97.548238574939
T1_3	45,885,508	6,916,627,657	97.039639614186	96.870397191707
T1_4	50,680,564	7,639,861,117	97.486593530478	97.322212217603
T1_5	46,009,384	6,937,787,414	97.669545009641	97.534168693133
T1_6	42,799,156	6,454,429,227	97.694728722182	97.57011622
T2_1	40,132,924	6,051,196,174	97.783047287389	97.639837959693
T2_2	45,378,666	6,839,966,373	97.268726194934	97.095370459131
T2_3	45,073,698	6,795,372,732	97.652654844537	97.498335196405
T2_4	42,958,040	6,475,499,338	97.67511257	97.506996335877
T2_5	40,191,344	6,059,936,489	97.497226310009	97.353340179849
T2_6	41,239,558	6,218,059,958	97.348251475418	97.205784936683
T3_1	47,458,224	7,153,173,391	96.90185459	96.72581824
T3_2	54,497,290	8,216,382,616	97.474396852991	97.323867271125
T3_3	47,920,768	7,226,232,745	97.688676477861	97.556329999164
T3_4	48,148,120	7,259,407,355	97.659908269527	97.512703580384
T3_5	46,894,746	7,069,674,326	97.776101335579	97.618243569173
T3_6	44,291,602	6,677,188,449	96.89977867	96.74267293
T4_1	43,375,150	6,539,364,932	97.161513753005	97.008973570729
T4_2	49,739,036	7,502,295,718	97.93891108	97.830694962009
T4_3	51,319,456	7,737,338,679	97.939156619654	97.788768235467
T4_4	45,918,430	6,924,185,704	97.894305072706	97.760216977133
T4_5	45,732,638	6,896,316,820	97.86211538	97.730158542206
T4_6	45,184,990	6,813,928,848	97.89557295	97.766374182185

**Table A3:** Post-host sequence statistics table.

Samples	Optimized Reads	Optimized Bases (bp)	Percent in Raw Reads (%)	Percent in Raw Bases (%)
T1_1	37,708,956	5,687,190,752	86.636169706828	86.531769000365
T1_2	40,606,218	6,123,659,514	87.786613994693	87.673802805413
T1_3	36,241,550	5,465,501,039	76.644393934998	76.546733864413
T1_4	42,225,562	6,367,892,544	81.222975326202	81.118933977352
T1_5	41,246,028	6,220,597,224	87.557807516287	87.451624388682
T1_6	37,487,584	5,654,498,476	85.570363801801	85.477747771693
T2_1	36,100,062	5,443,995,000	87.957061629093	87.842266945051
T2_2	38,005,580	5,730,882,551	81.464588555769	81.351593560987
T2_3	37,629,298	5,674,739,206	81.524281625089	81.419761222712
T2_4	36,332,484	5,479,001,223	82.610367340209	82.501892794623
T2_5	35,223,316	5,312,288,504	85.445652462903	85.34231849
T2_6	36,098,904	5,444,682,231	85.213454144658	85.115713513543
T3_1	39,217,648	5,913,757,392	80.075959522503	79.966329813763
T3_2	47,671,578	7,189,203,274	85.265860239662	85.156826054011
T3_3	42,259,594	6,373,865,458	86.148114453252	86.049113546913
T3_4	42,236,194	6,369,885,666	85.668616587604	85.564115969023
T3_5	40,421,382	6,095,564,496	84.279060655455	84.167710169303
T3_6	38,894,234	5,865,937,143	85.091586121776	84.988830670211
T4_1	32,835,434	4,952,365,077	73.55226373	73.466438692902
T4_2	42,530,456	6,416,293,352	83.744818627956	83.669114268614
T4_3	44,683,348	6,738,608,652	85.27466499	85.166265435482
T4_4	39,747,726	5,995,135,634	84.738873149416	84.64327582
T4_5	40,598,262	6,123,497,343	86.875194033139	86.778258856757
T4_6	37,815,316	5,704,297,584	81.928800383462	81.845364764495
